# The prototype device for non-invasive diagnosis of arteriovenous fistula condition using machine learning methods

**DOI:** 10.1038/s41598-020-72336-5

**Published:** 2020-10-02

**Authors:** Marcin Grochowina, Lucyna Leniowska, Agnieszka Gala-Błądzińska

**Affiliations:** 1 University of Rzeszów, Al. Rejtana 16c, 35-310 Rzeszów, Poland; 2 Dialysis Centre, St. Quenn Jadwiga Clinical District Hospital No 2 in Rzeszów, Lwowska 60 Street, 35-301 Rzeszów, Poland

**Keywords:** Software, Diagnosis

## Abstract

Pattern recognition and automatic decision support methods provide significant advantages in the area of health protection. The aim of this work is to develop a low-cost tool for monitoring arteriovenous fistula (AVF) with the use of phono-angiography method. This article presents a developed and diagnostic device that implements classification algorithms to identify 38 patients with end stage renal disease, chronically hemodialysed using an AVF, at risk of vascular access stenosis. We report on the design, fabrication, and preliminary testing of a prototype device for non-invasive diagnosis which is very important for hemodialysed patients. The system includes three sub-modules: AVF signal acquisition, information processing and classification and a unit for presenting results. This is a non-invasive and inexpensive procedure for evaluating the sound pattern of bruit produced by AVF. With a special kind of head which has a greater sensitivity than conventional stethoscope, a sound signal from fistula was recorded. The proces of signal acquisition was performed by a dedicated software, written specifically for the purpose of our study. From the obtained phono-angiogram, 23 features were isolated for vectors used in a decision-making algorithm, including 6 features based on the waveform of time domain, and 17 features based on the frequency spectrum. Final definition of the feature vector composition was obtained by using several selection methods: the feature-class correlation, forward search, Principal Component Analysis and Joined-Pairs method. The supervised machine learning technique was then applied to develop the best classification model.

## Introduction

An arteriovenous fistula (AVF) is an abnormal, artificial connection between a vein and an artery which allows access to the vascular system. This permanent vascular access provides a sufficient blood flow for hemodialysis—a life-sustaining treatment removing waste products and excess fluid from the blood of an end-stage renal disease^1^.

A patient and dialysis staff must take great care to prevent AVF functional failure. Adequate renal replacement therapy and reduced mortality in this population depend on the proper functioning of AVF^2,3^.

Hence, once the AVF is developed, it needs to be monitored periodically to ensure that the fistula can manage the process of hemodialysis. The main objective is to observe the development and progression of stenosis (vascular narrowing) which increases the risk of thrombosis (vascular occlusion caused by clotting). Such detection is a worldwide public health problem that should be managed in its early stages.

It is well known that stenosis in the artery causes a swishing sound, which is heard as a bruit (pathological blood flow sound) during an auscultation. The first mention of the numerical analysis of acoustic signals emitted by the AVF occurred in the mid-1980s^4^. In 1996, Bosman^5^ found a dependence in the time domain when the effect of the fistula’s state on the amplitude of the emitted sound was analysed. In the following years, the phono-angiography as a non-invasive method for evaluating the acoustic noise emitted from the vessel produced by the local blood flow through the vascular system was intensively explored. The detailed overview of these issues was presented by Noor^6^.

Listening for bruits during a physical examination is a subjective test. This means that vascular access dysfunction requires high skills and experience or dedicated tools that are accurate and reliable. The literature contains various more precise methods for detecting vascular access stenosis. Some of them, like duplex Doppler ultrasound scanning^7^, or magnetic resonance (MR) angiography^8^ provide an accurate indication of the degree of vascular access stenosis. However, they involve certain surgical risks (i.e. while using contrast) and require the use of expensive apparatus, hence, they are rather impractical for an initial screening in hemodialysis centres. Therefore, uncomplicated but effective ways of assessing the condition of the fistula are still being sought^9^.

The methods of automatic assessment of arteriovenous fistula condition described so far in the literature are mainly based on a two-class classification, dividing the fistula into “good” and “bad”. Few attempts were made for multi-class assessment, divided into, for example, 3 classes^10^—good, medium and bad. From the point of view of patient well-being, it is important to be able to detect adverse changes early, which is impossible for two classes, and at least not very precise for three classes.

In addition, no attempts have been made to produce an easily available technical solution that allows constant observation of AVF, while devices of this type in other diagnostic areas are publicly available. For example, telemonitoring of people affected by heart disease^11^ or epilepsy^12^ is common.

Since the main objective for patients on hemodialysis is to observe the progression of stenosis over time to ensure that the fistula can manage the process of hemodialysis, it is crucial to develop a low-cost tool detecting a degree of vascular access stenosis. It can be done with the phono-angiography technique and a machine-learning approach. The acquisition of a large amount of data and the choice of an effective classifier enable automation of decision-making processes in medical diagnostics. The general concept is to recognize objects based on patterns and assign them to appropriate classes. To the authors’ knowledge, up to date, the need for fully automated devices offering AVF condition assessment has not been satisfied.

The aim of this work is to develop a low-cost tool for monitoring AVF with the use of phono-angiography method. This is a non-invasive and inexpensive procedure for evaluating the sound pattern of bruit produced by AVF. With a special kind of head^13^ which has a greater sensitivity than conventional stethoscope, a sound signal from fistula was recorded. The process of signal acquisition was performed by a dedicated software, written specifically for the purpose of our study. From the obtained phono-angiogram, 23 features were isolated for vectors used in a decision-making algorithm, including 6 features based on the waveform of time domain, and 17 features based on the frequency spectrum. Final definition of the feature vector composition was obtained by using several selection methods: the feature-class correlation^14^, forward search^15^, Principal Component Analysis (PCA)^16^ and Joined-Pairs method^17^. The supervised machine learning technique was then applied to develop the best classification model.

## Material and methods

### Materials

The research material was collected from 38 patients on dialysis at the Clinical Dialysis Centre of the St. Queen Jadwiga Provincial Hospital No. 2 in Rzeszow. The study was approved by the local Bioethical Committee of the Regional Medical Chamber in Rzeszow (No. 17/B/2016). The data collection and all experiments were performed in accordance with the relevant guidelines and regulations. All patients included in the study have been informed about its course and purpose, and they gave written informed consent to participate in the study. Selected clinical and demographic data of the study group are presented in Table 1.

**Table 1:** Selected clinical and demographic data of the study group.

Variable	$\hbox {Mean} \pm \hbox {SD}$ (min–max)
Age [years]	$64.74 \pm 15.29$ (18–29)
Sex; female [n ( %)]	13 (34.2)
BMI [kg/m2]	$25.94 \pm 6.81$ (16.06–44.87)
Total dialysis time [months]	$66.43 \pm 26.86$ (19–132)
Serum creatinine before hemodialysis [umol/L]	$645.3 \pm 527.8$ (335.9–1255.3)
Smoking [n ( %)]	11 (28.9)
Type 2 diabetes [n ( %)]	20 (52.6)
Cardiovascular diseases	21 (55.3)
A history of stroke	13 (34.2)

The registration process was carried out just before subjecting patients to dialysis, which allowed to ensure a high degree of repeatability of the physiological state of patients, in particular the level of hydration of the body affecting the blood density. A single room, isolated from other rooms of the ward by a double door, ensured separation from external acoustic interference. The sampling rate was set at 8 kS/s with a resolution of 16 bit/sample. In total, while recording in several series, 156 recordings were obtained, from which 2670 feature vectors were extracted. Patients were divided due to their physiological state, based on the opinion of medical staff, ranked from the best to the worst.

All cases were therefore divided into six sub-classes from ’patent’ (A) to ’failed’ (F), proposed by the medical staff of dialyze centre, as 6 recognizable and successive states of deteriorating of AVF functions. The best cases were labelled A, while the worst cases were labelled F. The assignment of individual patients to specific classes was made based on the the results of ultrasound imaging tests. The number of patients assigned to each class and the number of feature vectors obtained from each patient is presented in Table 2.

**Table 2:** Number of patients and vectors in classes denoting cases with an increasing degree of a-v fistulas pathologisation assessed on the basis of Doppler ultrasound (*A* proper AVF function; *F* limited flow in the AVF).

Class	A	B	C	D	E	F
Patients per class	3	5	7	10	9	4
Vectors per class	232	348	516	692	669	251
Vectors per patient	67	86	75	82	90	74
	73	60	57	62	95	60
	89	53	80	70	73	60
		55	59	59	66	53
		89	87	77	64	
			96	58	84	
			55	72	72	
				57	61	
				59	55	
				86		

Classes were determined on the basis of mathematical analysis of the location of samples in the space of cases. Each class is a cluster of closely located samples. The order of the classes was determined on the basis of the mutual location of the classes in the case space, i.e. the distance of classes A and B is smaller than the distance of classes A and C, which in turn is smaller than A and D, etc. The order of classes determined in this way was confirmed by the assessment of the clinical state of the fistulas by medical staff. The number of six classes was the largest for which total compliance of the mathematical model with clinical assessment was obtained.

**Figure 1:**
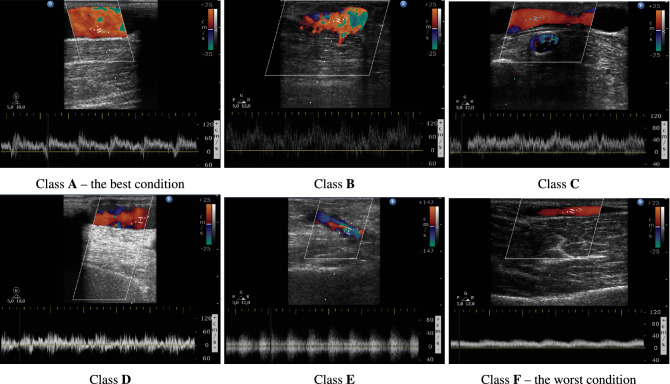
Image from fistula USG examination in six stages of its function. The letters and numbers of Figures correspond to the AVF functional classes presented in Table 1.

The Fig. 1 shows sample images of an ultrasound of fistulas in progressive pathologization. The technique used to determine the flow parameters based on the Doppler effect was used. The upper part of the image shows a map of colours velocity and the direction of blood flow through the vessel fistula marked with selected measuring points. The lower part shows a time diagram of instantaneous values of the speed and direction of the flow measured at the point marked with a marker. The quality of the AVF was assessed by Dialysis Center doctors on the basis of a Doppler ultrasound image, the quality of blood supply from the AVF to the dialyzer during the hemodialysis and degree of excretion from urea during the hemodialysis.

### Features extraction

**Figure 2:**
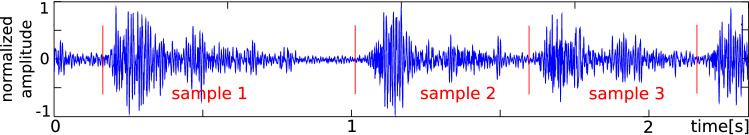
Example of division of the recorded signal from arteriovenosu fistula into fragments corresponding to single heartbeats.

A 30-second signal, containing not less than 20 heart cycles, was recorded each time. The recorded signal was divided into fragments corresponding to individual heart cycles (Fig. 2). The division points were indicated by the local envelope minima, and each fragment obtained in this way was used to create one vector of features. Frequency domain analysis was performed using Fast Fourier Transform (FFT). Because the input signal was sampled at 8 kS/s, an FFT of 8192 points was used, resulting in a spectrum resolution of 1Hz.

The length of a single segment signal depends on the pulse. While analysis using FFT requires the packets of predetermined length, the packets longer than 8192 points were cut into 8192 points and shorter packets supplemented to the required length with zeros.

In the course of research, it was found that the type of window used did not affect the final classification results, therefore a rectangular window was used. For further analysis, only the FFT module was standardized according to the formula:

1 $$\begin{aligned} X_N[i] = \frac{X[i]}{\sum _{n=0}^{8191}X[n]}, \end{aligned}$$

while the values of the features were calculated according to the formula:

2 $$\begin{aligned} T_f = \sum _{n=f_{dt}}^{f_{gt}}X_N[n] \end{aligned}$$

where $T_f$—features value, $f_{dt}, f_{gt}$—lower and upper third border frequency *f*.

### Features selection

The final set of diagnostic features to be implemented was selected based on an in-depth analysis of classification quality indicators and the assignment of the examined vectors to individual classes. Preliminary estimations of the classification value of each feature were made using the following selection methods: feature-class Correlation, Forward Search, PCA and Joined-Pairs. The last method was used to assess the classification quality of pairs of features and was created in the course of work on developing a classification system for arteriovenous fistula^17^.

Selection methods from the wrapper group (Forward Search and Joined-Pairs) require the use in the ranking process of the classifier which will be ultimately used in the system under construction. The order of the features in the ranking is determined by the quality of the classification achieved using subsets of the features considered. Assessment of classification quality, in turn, requires the division of the data set into the teaching and test part. Typically, n-fold cross-validation is used in such cases. In our case 10-fold validation was used for feature ranking. Very high indices of quality classification (accuracy up to 0.95) were achieved. However, in the process of verifying results using feature vectors from new patients whose samples were not part of the original data set, significant deviations in the operation of the system were obtained. It was found that the classification system learned the individual characteristics of patients rather than the generalized state of their AVFs. For this reason, modifications were made to the method of splitting the data set into training and test subsets, and leave-one-out cross-validation was used—a subset of one patient was excluded as a test set in each iterative course of validation. This allowed the study to avoid undesirable impactsfrom the tested and training set correlations, which could overestimate the classification quality indicators. Examples of feature rankings for the selection methods used are presented in Table 3. The names of $T_f$ refer to features calculated using formula (2).

**Table 3:** Ranking of features for standard and leave-one-out cross-validation.

No	Correlation	10-fold		Leave-one-out	
		Cross-validation		Cross-validation	
		Forward search	Joined pairs	Forward search	Joined pairs
1	$T_{400}$	$T_{31}$	$T_{31}$	$T_{315}$	$T_{315}$
2	$T_{500}$	$T_{315}$	$T_{315}$	$T_{500}$	$T_{500}$
3	$T_{100}$	$T_{200}$	$T_{200}$	$T_{100}$	$T_{400}$
4	$T_{630}$	$T_{160}$	$T_{20}$	$T_{80}$	$T_{250}$
5	$T_{80}$	$T_{40}$	$T_{250}$	$T_{400}$	$T_{125}$
6	$T_{63}$	$T_{20}$	$T_{40}$	$T_{630}$	$T_{80}$
7	$T_{315}$	$T_{400}$	$T_{125}$	$T_{125}$	$T_{100}$
8	$T_{125}$	$T_{500}$	$T_{25}$	$T_{63}$	$T_{630}$
9	$T_{50}$	$T_{630}$	$T_{80}$	$T_{250}$	$T_{200}$
10	$T_{40}$	$T_{125}$	$T_{50}$	$T_{50}$	$T_{31}$
11	$T_{160}$	$T_{100}$	$T_{160}$	$T_{200}$	$T_{25}$
12	$T_{250}$	$T_{80}$	$T_{100}$	$T_{31}$	$T_{49}$
13	$T_{200}$	$T_{250}$	$T_{400}$	$T_{160}$	$T_{160}$
14	$T_{31}$	$T_{25}$	$T_{500}$	$T_{40}$	$T_{63}$
15	$T_{20}$	$T_{50}$	$T_{630}$	$T_{25}$	$T_{50}$
16	$T_{25}$	$T_{63}$	$T_{63}$	$T_{20}$	$T_{20}$

The Table 3 contains the ranking of features obtainedusing the Correlation method and the Forward search and Joined-Pairs methods in two variants—using 10-fold cross-validation and leave-one-out cross-validation for the k Nearest Neighbours (k-NN) classifier.

**Figure 3:**
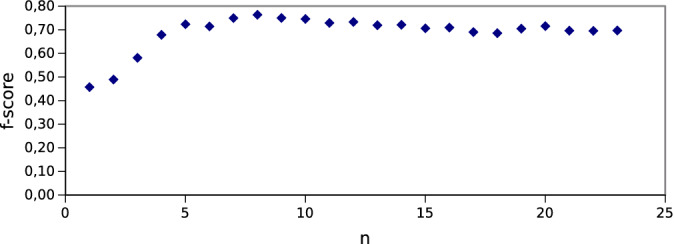
F-score as a function of the number of features (k-NN classifier); features added in the order indicated by ranking of the Correlation method.

The selection of the final set of features was based on the assessment of the classification ability of the subsets that were built by successively adding features in the order of ranking. In this way, each subset was checked for each ranking and for each of the classifiers used. Figure 3 shows the value of the average F-score indicator as a function of the number of features in the set for subsets defined by the Correlation method. Thus, the subsequent points of the graph from the left illustrate the value of F-score for the sets of features $\{T_{400}, T_{500}\}$, $\{T_{400}, T_{500}, T_{100}\}$, $\{T_{400}, T_{500}, T_{100}, T_{630}\}$ etc. It is worth noting that the first 8 features indicated by the Correlation method coincide with the first eight features indicated by the Forward Search method, though in a different order (Table 3). In addition, a subset of 8 features maximizes the average value of the F-score at around 0.76. This is the highest value achieved when using leave-one-out cross-validation.

Analysis of the selection results showed features describing the energy content in two frequency bands: 60–140 Hz and 280–700 Hz. With reference to specific features calculated on the basis of thirds, it indicated features $T_{63}, T_{80}, T_{100}, T_{125}, T_{315}, T_{400}, T_{500}$ i $T_{630}$ and these 8 features established the basis for further construction of the classification system.

### Classification system

The selection of the classification algorithm was based on empirical studies of the classification quality, obtained for k-NN, Support Vector Machine (SVM) and Random Forest algorithm classifiers^18^. The work parameters of classifiers were selected in the iterative process of searching for optimal values^19^. The best quality indicators for each classifier were calculated for each class separately based on confusion matrixes. and presented in the Tables 4, 5 and 6. The values in the confusion matrixes were expressed as a percentage.

**Table 4:** Confusion matrix and classification quality indicators—k-NN classifier.

Class	a	b	c	d	e	f
a $\rightarrow$	91.0	9.0	0.0	0.0	0.0	0.0
b $\rightarrow$	9.0	69.0	22.0	0.0	0.0	0.0
c $\rightarrow$	0.0	23.0	67.0	10.0	0.0	0.0
d $\rightarrow$	0.0	0.0	4.0	81.0	15.0	0.0
e $\rightarrow$	0.0	0.0	0.0	9.0	83.0	8.0
f $\rightarrow$	0.0	0.0	0.0	0.0	8.0	92.0
Precision	0.91	0.68	0.72	0.81	0.78	0.92
Recall	0.91	0.69	0.67	0.81	0.83	0.92
F-score	0.91	0.69	0.69	0.81	0.81	0.92
Acc	0.81

**Table 5:** Confusion matrix and classification quality indicators—SVM classifier.

Class	a	b	c	d	e	f
a $\rightarrow$	88.0	12.0	0.0	0.0	0.0	0.0
b $\rightarrow$	10.0	65.0	25.0	0.0	0.0	0.0
c $\rightarrow$	0.0	26.0	64.0	10.0	0.0	0.0
d $\rightarrow$	0.0	0.0	5.0	79.0	16.0	0.0
e $\rightarrow$	0.0	0.0	0.0	10.0	81.0	9.0
f $\rightarrow$	0.0	0.0	0.0	0.0	10.0	90.0
Precision	0.90	0.63	0.68	0.80	0.76	0.91
Recall	0.88	0.65	0.64	0.79	0.81	0.90
F-score	0.89	0.64	0.66	0.79	0.78	0.90
Acc	0.78

**Table 6:** Confusion matrix and classification quality indicators—Random Forest classifier.

Class	a	b	c	d	e	f
a $\rightarrow$	86.0	14.0	0.0	0.0	0.0	0.0
b $\rightarrow$	13.0	61.0	26.0	0.0	0.0	0.0
c $\rightarrow$	0.0	27.0	60.0	13.0	0.0	0.0
d $\rightarrow$	0.0	0.0	7.0	75.0	18.0	0.0
e $\rightarrow$	0.0	0.0	0.0	13.0	76.0	11.0
f $\rightarrow$	0.0	0.0	0.0	0.0	12.0	88.0
Precision	0.87	0.60	0.65	0.74	0.72	0.89
Recall	0.86	0.61	0.60	0.75	0.76	0.88
F-score	0.86	0.60	0.62	0.75	0.74	0.88
Acc	0.74					

The data presented in the tables show that the best quality parameters were obtained using the k-NN algorithm with the Manhattan metric and distance-weighted voting, with $k = 7$. The highest quality indicators were achieved both for the classifier in general and for each class separately. For this reason, the k-NN classifier has been selected for the implementation in the target environment.

## Implementation

### System concept

**Figure 4:**
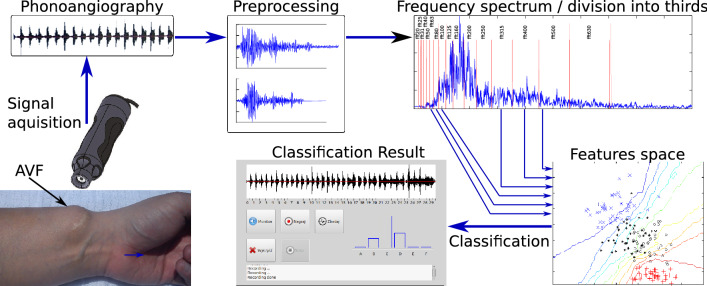
System concept.

The studies mentioned above have shown that the phono-angiogram taken from AVF can be used to evaluate occluded blood vessels. Many patients who would be at-risk for stenosis progression definitely need an easy to operate tool for checking the AVF condition. For this purpose, we have created a system called ’NefDiag’ for detecting vascular access stenosis by means of a simple phono-angiography procedure implemented on a microprocessor device and equipped with a dedicated application for predictive stenosis progression with 80% accuracy. The idea was summarized in the Fig. 4.

### Hardware

**Figure 5:**
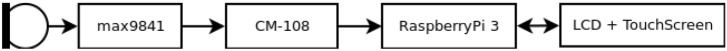
The structure of the hardware layer.

The basis for choosing the hardware platform was the possibility to run solutions developed during the research process and the implementation of the program layer indicated in the assumptions, as well as the provision of mobility and the ergonomics of use by the potential user. All these requirements were met by a microcomputer designed for embedded applications from the Raspberry Pi series. A version III based on a 64-bit 4-core ARM processor equipped with a floating-point unit was chosen. The block diagram of the hardware layer is shown in Fig. 5.

To build the head recording the acoustic signal from the arteriovenous fistula, an external USB sound card and integrated mic preamplifier were used. The card is based on the C-media CM-108 system and the preamplifier is max9841. As the GUI basis, a 7” LCD display with a touch panel was used.

### Software

The choice of technology for the implementation of the programming layer of the designed system was performed in two stages. First, the ease of use of the operating system and its type were considered. Next, the technology for the implementation of algorithms of data processing and user interaction were selected.

It was initially assumed that the diagnostic system would be created without the support of the operating system. However, it was found that building the application directly on the hardware layer will shift the centre of gravity of the implementation to software-hardware communication and will build the middle layer between the target application and the microprocessor and its peripherals. Available hardware platforms can run operating systems that provide hardware support tools through convenient interfaces, allowing developers to focus on constructive coding. The possibilities of using Linux, Windows and RTOS were considered.

RTOS was rejected due to the lack of hardware layer drivers and the need to re-implement any developed processing algorithms and data classification. Windows, despite very convenient development tools, does not allow easy connection and transfer of data between modules that use different programming technologies, in particular different languages. In addition, it is a commercial licensed system, which leads to financial consequences. As a result, Linux was chosen, primarily due to being well known by members of the research team, and because of a number of possible programming techniques that are impossible or difficult to obtain via other systems.

**Figure 6:**
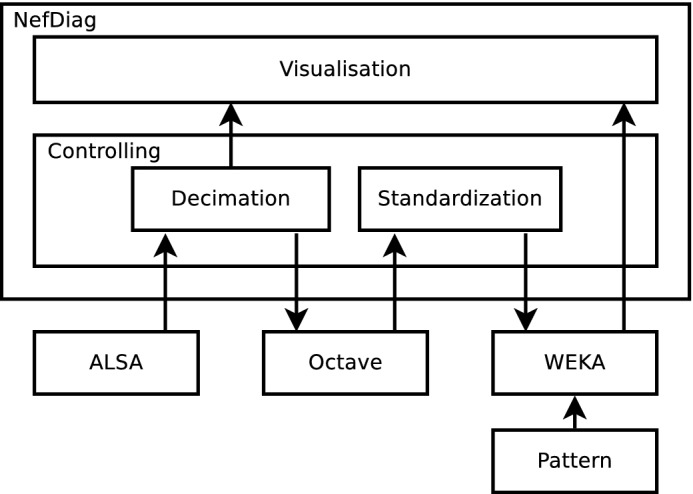
The structure of the software layer.

The selected operating system indicated a way to implement the diagnostic application. Since the entire research process was carried out using tools working under the control of the Linux operating system, it was possible to reuse them, this time in the target system. Therefore, a modular system structure was adopted based on the master module responsible for data flow and control, and executive modules responsible for individual elementary operations, not necessarily made using the same techniques and programming languages as the main module (Fig. 6).

The master module, responsible for the control flow, data distribution and performing the role of the user interface, was written in the C++ language using the Qt library. Modules performing audio acquisition and data analysis tasks used ready-made solutions available in the form of programming libraries or computing environments. The whole was connected through glue logic scripts.

Audio acquisition was done using the ALSA (Advanced Linux Sound Architecture) library that provides communication with the hardware layer, data buffering, and error handling. Due to the hardware limitations of the sound card used (44 100 or 48 000 S/s sampling rate), it was necessary to decimate the signal to the required sampling rate of 8 kHz.

The signal analysis module was designed in the form of a script executed under the control of the Octave package. It successively divides into fragments corresponding to the heart rhythm and extracts diagnostic features based on the FFT. The next step is classification using the WEKA package and visualization the results on the LCD.

The diagnosis is presented in the form of a bar chart on the LCD display.

### The diagnostic device

**Figure 7:**
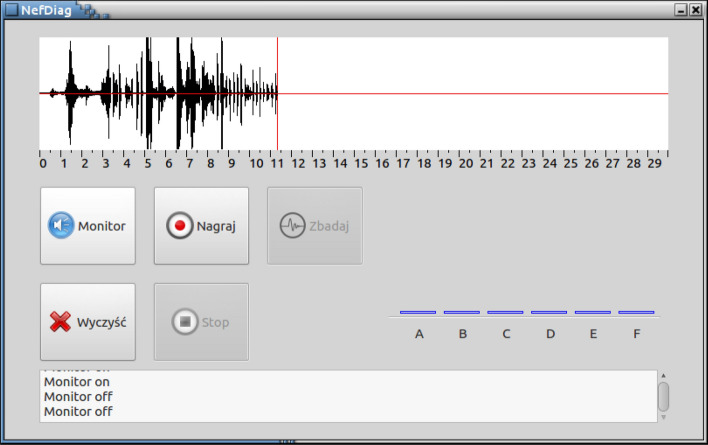
NefDiag application control panel.

The user interface consists of four sections (Fig. 7):

- graph of the time history of the recorded sound signal,
- control panel with buttons,
- bar chart presenting the test result,
- notification area.

The time chart makes it possible to observe the graphical representation of the signal during recording, which allows the operator to choose where the head should be applied to the patient’s body in such a way that the signal is of the best possible quality with a clear heart rhythm and without any distortion.

In the control panel, one of the five operating options for the device can be chosen:

Monitor: continuous signal registration with visualization in the field of the time graph, enabling selection of the optimal head application site for the fistula,
Record: registering a 30-s recording for analysis,
Analyze: starting the process of analysing the recorded signal,
Clear: flush buffers and reset user interface before performing next test,
Stop: stopping the Monitor or the Record function.

**Figure 8:**
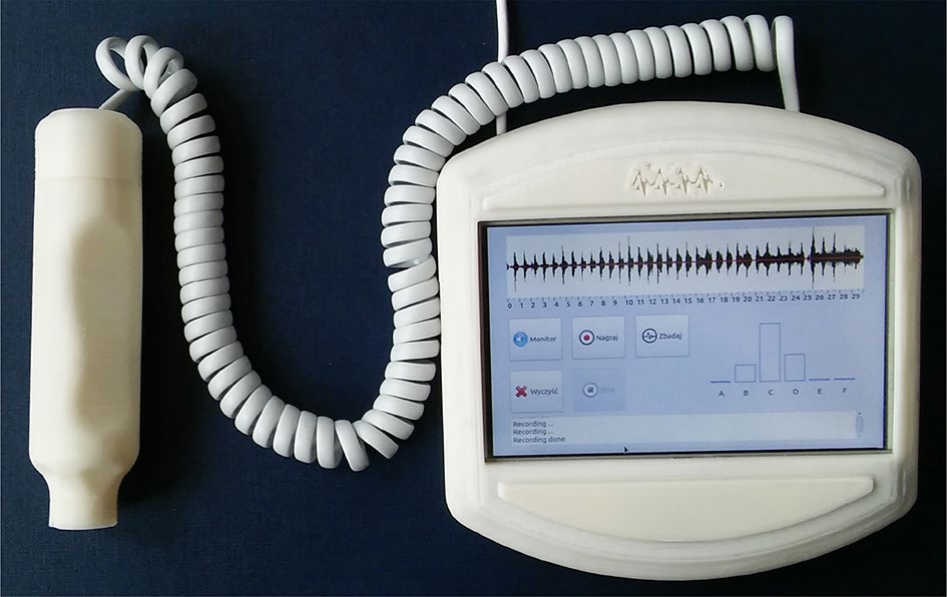
NefDiag device.

The test result is presented in the form of a bar chart. The height of each bar represents the percentage probability of assigning the test result to one of the six classes describing the state of the fistula. The device was enclosed in a dedicated cover made with rapid prototyping with the use of a 3D printer (Fig. 8). The part responsible for A/C processing (including the microphone, preamplifier and sound card) has been made as a separate mechanism and connected to the device via a USB cable. Preliminary tests with patients have confirmed the correct operation of the device.

**Figure 9:**
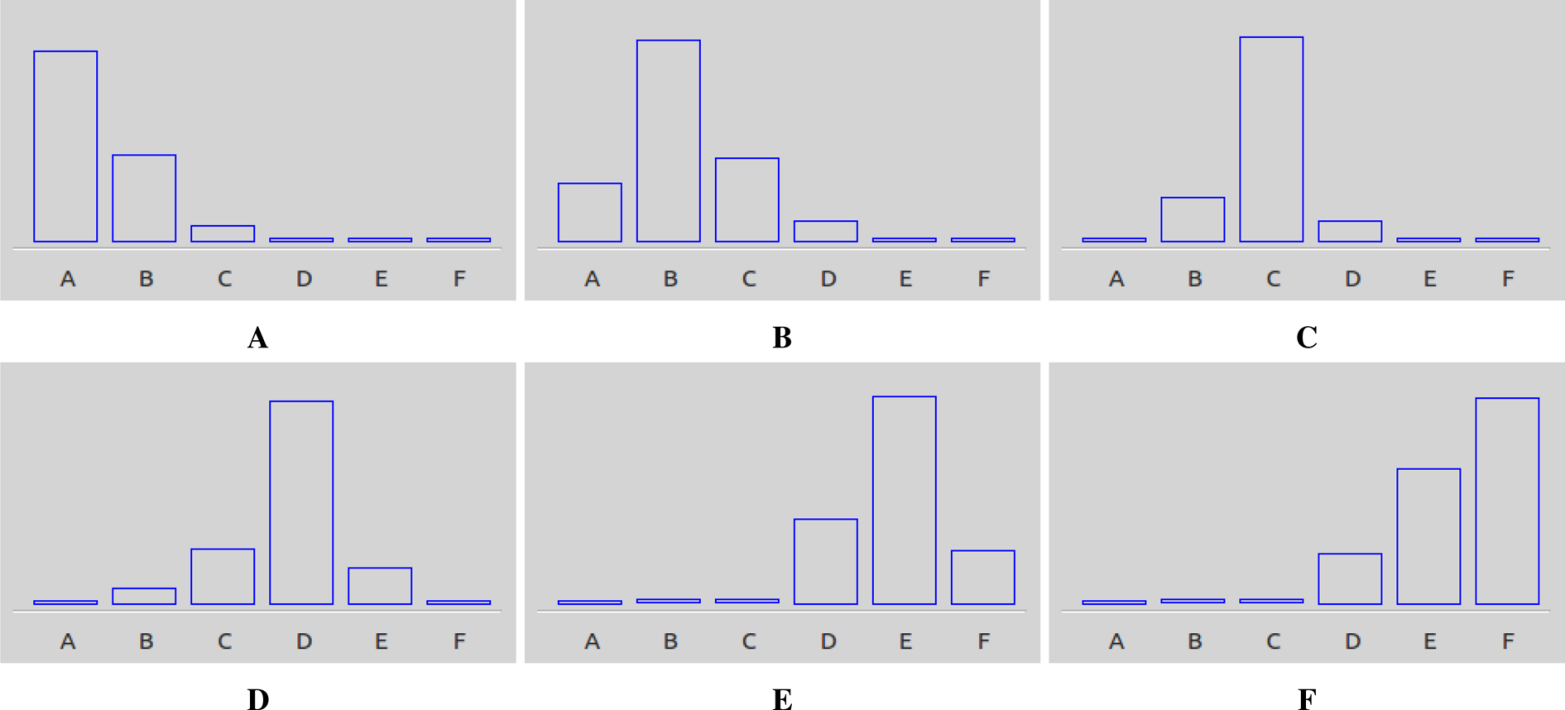
Indications of the device in response to the fistula signal at various stages of pathologization.

Figure 9 presents examples of device indications in response to signals from fistulas in various states. The highest bar of the graph indicates the class in which the fistula was classified. The lateral, lower bars are the result of noise and interference in the signal causing erroneous recognition of some samples and assigning them to neighboring classes. The cases whose results are depicted in Fig. 9 show the classification results for the cases whose ultrasound images are presented in Fig. 1.

## Discussion

In our study, we have described an AVF condition assessment system, based on the results of acoustic signal measurements acquired with an electronic head specifically designed for this task. The proposed low-cost device was created using a machine-learning approach. The general concept was to recognize patients with significant stenosis based on patterns developed and assign them to appropriate classes. This activity was performed in a classical way, as a sequence of successive operations: feature extraction, selection and reduction of features, classification, and interpretation of the classification results. As a final achievement we have constructed a prototype of microprocessor device with the intelligent assessment system which based on the six-step patient classification (letters A-F, from the best condition to the worst).

Based on available literature, up to date, the need for fully automated devices offering the AVF condition assessment has not been satisfied. Both patients and doctors need an easy-to-use and reliable support system in assessing the condition of the fistula. To the best of the authors’ knowledge, there is no such tool yet. The proposed device based on machine learning technique can be such support.

Due to the limited number of collected cases forming the classification system, diagnostic indications have been discretized in the form of six classes. The sequence of disease states, from ‘patent’ (A) to ‘failed’ (F), was determined on the basis of mathematical analysis of the location of samples in the space of cases and confirmed by the medical staff of dialyze cente, as 6 recognizable and successive states of deteriorating of AVF functions. The number of six classes was the largest for which an unambiguously consistent assessment of the AVF status by medical personnel was obtained.

Experiment results show that the acustic signal from a single AVF is classified in the correct, expected class with very good accuracy of 81%. Furthermore, it has been observed that classification by these methods correlates with the results of the classic diagnostic tests used to evaluate AVF functioning, such as Doppler USG. An important result of this study is the confirmation of the possibility of obtaining a quick, non-invasive and reliable diagnosis of the AVF condition, as well as the solution of technological problems toward a fully automated assessment. Finally, it can be stated that the low-cost device created can help doctors and patients with renal disease to assess/predict stenosis progression due to cell growth by providing an easy to use and high accuracy tool.

The strength of this research is finding a trade-off between how much information we can extract from a single measurement vs. how reliable that information is. The 6th grade, medically justified scale used (see Fig. 1) seems to be accurate enough for diagnostic purposes, while giving a high probability of a correct, non-subjective assessment of the state of the AVF. The limitations of our study are a small number of patients studied, the use of AVF division into functional classes based on the experience of dialysis station staff.

The performance of the described method can be greatly degraded in noisy environments. By only using spectral information it can be hard sometimes to directly distinguish the dynamic characteristics of bruit directly from a noisy phono-angiography. However, the doctors with experience can recognize the stenosis sound pattern even in the noisy environments. Therefore, we are going to expand the device menu and add some further options with possibility to assess AVF condition with the use of different methods, like wavelet transform^20,21^ or others proposed in the literature for detecting hemodynamically significant vascular access stenosis^9,22,23^.

## Conclusion

The acquisition of a large amount of data and the choice of an effective classifier enable automation of decision-making processes in medical diagnostics. In this paper, we have described an AVF condition assessment system, based on the results of acoustic signal measurements acquired with an electronic head specifically designed for this task. The proposed low-cost device was created using a machine-learning approach. The general concept was to recognize patients with significant stenosis based on patterns developed and assign them to appropriate classes. This activity was performed in a classical way, as a sequence of successive operations: feature extraction, selection and reduction of features, classification, and interpretation of the classification results. Taking into consideration that the proper selection of a set of features can significantly improve the quality of the classification process, we compared several methods of feature selection. We also compared the quality of classifications using SVM, k-NN and Random Forest classifiers and demonstrated the possibility and relevance of the multi-class approach. All operations mentioned above were performed on the recorded acoustic signals obtained from 38 patients on chronic hemodialysis, without any adjustments for the prior medical conditions, age, gender, etc.

The key element of this process was the classification that transforms the vector of features describing a given sample into a value representing one of the possible object classes. It is worth emphasizing that the sequence of disease states (from ’patent’ (A) to ’failed’ (F)) was proposed by the medical staff of dialysis centre as 6 recognizable and successive states of deteriorating of AVF functions.

Finally, it can be stated that the low-cost device created can help doctors and patients with renal disease to predict AVF fistula stenosis progression by providing an easy to use and high accuracy tool. Up to date, the need for fully automated devices offering the AVF condition assessment has not been satisfied.
